# Multisite Phosphorylation Provides an Effective and Flexible Mechanism for Switch-Like Protein Degradation

**DOI:** 10.1371/journal.pone.0014029

**Published:** 2010-12-13

**Authors:** S. Marjan Varedi K., Alejandra C. Ventura, Sofia D. Merajver, Xiaoxia Nina Lin

**Affiliations:** 1 Department of Chemical Engineering, University of Michigan, Ann Arbor, Michigan, United States of America; 2 Comprehensive Cancer Center, University of Michigan, Ann Arbor, Michigan, United States of America; 3 Center for Computational Medicine and Bioinformatics, University of Michigan, Ann Arbor, Michigan, United States of America; 4 Department of Biomedical Engineering, University of Michigan, Ann Arbor, Michigan, United States of America; Fondazione Telethon, Italy

## Abstract

Phosphorylation-triggered degradation is a common strategy for elimination of regulatory proteins in many important cell signaling processes. Interesting examples include cyclin-dependent kinase inhibitors such as p27 in human and Sic1 in yeast, which play crucial roles during the G1/S transition in the cell cycle. In this work, we have modeled and analyzed the dynamics of multisite-phosphorylation-triggered protein degradation systematically. Inspired by experimental observations on the Sic1 protein and a previous intriguing theoretical conjecture, we develop a model to examine in detail the degradation dynamics of a protein featuring multiple phosphorylation sites and a threshold site number for elimination in response to a kinase signal. Our model explains the role of multiple phosphorylation sites, compared to a single site, in the regulation of protein degradation. A single-site protein cannot convert a graded input of kinase increase to much sharper output, whereas multisite phosphorylation is capable of generating a highly switch-like temporal profile of the substrate protein with two characteristics: a temporal threshold and rapid decrease beyond the threshold. We introduce a measure termed temporal response coefficient to quantify the extent to which a response in the time domain is switch-like and further investigate how this property is determined by various factors including the kinase input, the total number of sites, the threshold site number for elimination, the order of phosphorylation, the kinetic parameters, and site preference. Some interesting and experimentally verifiable predictions include that the non-degradable fraction of the substrate protein exhibits a more switch-like temporal profile; a sequential system is more switch-like, while a random system has the advantage of increased robustness; all the parameters, including the total number of sites, the threshold site number for elimination and the kinetic parameters synergistically determine the exact extent to which the degradation profile is switch-like. Our results suggest design principles for protein degradation switches which might be a widespread mechanism for precise regulation of cellular processes such as cell cycle progression.

## Introduction

One third of all proteins in eukaryotic cells are phosphorylated at any time [1]. Phosphorylation profile has been interpreted as a “molecular barcode” [2] to direct protein for other processes such as activation, inactivation, translocation, and degradation. In particular, three major transitions in the cell cycle, namely, entry into the S phase, separation of sister chromatids, and exit from mitosis, involve degradation of certain proteins after phosphorylation-dependent ubiquitination [3]–[5]. Phosphorylation-driven ubiquitination through the SCF pathway and subsequent proteasomal degradation have been considered as a biochemical switch crucial for coordinating phase changes during the cell cycle [6], [7]. For instance, the ubiquitination and degradation of Sic1, a cyclin-dependent kinase inhibitor in yeast, and its functional homolog in mammalian cells, p27, are triggered by phosphorylation [8]–[11]. Degradation of Sic1 and p27 leads to the release of cyclins required for DNA synthesis in the S phase. Reduced nuclear p27 is observed in up to 60% of primary human breast cancers, which has been correlated with increased activity of the Src kinase family [12].

The number of phosphorylation sites observed in one protein can vary from 1 to over 100 [13]. It has become increasingly apparent that multisite phosphorylation is a widespread phenomenon among regulatory proteins in eukaryotic cells. Multisite protein phosphorylation potentially provides a precise tool for dynamic regulation of the downstream process. Different phosphorylation profiles of a single protein might be linked to different functions. For example, the retinoblastoma protein (Rb) has 16 Ser/Thr-Pro phosphorylation sites and interacts with different proteins during various cell cycle phases depending upon its phosphorylation profile [14], [15]. Most notably, in early G1, Rb is hypophosphorylated and sequesters the E2F family of transcription factors, thereby preventing the transcription of genes required for S-phase entry; while in late G1, Rb becomes hyperphosphorylated and thus inactive in repressing G1/S transition [16]. As another example, two sites in *Xenopus* protein Wee1A are responsible for the mitotic inactivation of this protein, while at least two others regulate its proteolysis during interphase [17]. Intriguingly, as far as protein stability is concerned, an alternative property of multisite phosphorylation, the degree of phosphorylation (i.e. the number of phosphate groups on a protein), instead of the exact pattern, might determine the protein's fate. A well-studied example is protein Sic1, which plays a key role in regulating the G1/S transition in the cell cycle of *Saccharomyces cerevisiae*.

Sic1 inhibits Clb5,6-Cdc28 kinase required for DNA replication and is believed to provide precise timing for the G1 to S transition by undergoing switch-like proteasome-mediated degradation upon phosphorylation by the Cln2-Cdc28 kinase complex. Yeast strains lacking Sic1 initiate DNA replication earlier and show extended S phase [18], [19]. On the other hand, in mutant strains that are resistant to Sic1 degradation, cells experience lengthened G1 phase in an otherwise wild-type genetic background [20] or G1 phase arrest in more complex situations [8], [21]. Sic1 is phosphorylated by the Cln2-Cdc28 kinase complex on nine Ser/Thr-Pro residues [8]. Nash *et al.* investigated how multisite phosphorylation of Sic 1 regulates its ubiquitination and degradation [9]. They began with the Sic1 mutant which lacks all the nine phosphorylation sites and restored the sites one by one in the order of their importance measured by the degree to which elimination of a single site affects the Sic1 turnover. Serial reintroduction of five sites failed to reestablish Sic1 binding to Cdc4, a subunit in the ubiquitin ligase 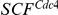 that determines the target specificity, or cell viability. Astonishingly, re-addition of a sixth seemingly insignificant site abruptly restored Sic1's binding with Cdc4 *in vitro* and revived the cells *in vivo*. These experiments clearly revealed that there is a threshold number of phosphorylated sites required to render binding of Sic1 with Cdc4. The “counting” mechanism underlying this multisite-dependent digital interaction between phosphorylated Sic1 and Cdc4 has been studied both theoretically and experimentally. Mathematical modeling suggested that cooperative interactions between a disordered multi-phosphorylated Sic1 and a single-site receptor Cdc4 can explain the observed phosphorylation threshold [22]. Furthermore, cumulative electrostatic forces resulted from negatively charged phosphate groups were proposed as the physical basis for the digital interaction between Sic1 and Cdc4 [23]. Recently, NMR analysis showed that Sic1 indeed exists in an intrinsically disordered state and its multiple phosphorylated sites interact with the single receptor site of Cdc4 in dynamic equilibrium [24], [25].

Upon their remarkable discovery that Sic1 requires at least six sites phosphorylated to bind to Cdc4 for subsequent ubiquitination and degradation, Nash *et al.* hypothesized that this phosphorylation threshold eventually causes Sic1 to degrade in a switch-like manner during the G1/S transition [9]. Reviewing the above seminal work, Deshaies and Ferrell conjectured more specifically that multisite phosphorylation can create temporal thresholds [26]. They calculated time courses for Sic1 destruction in three scenarios: Sic1 destruction triggered by one fast, one slow, or six fast phosphorylations. It was suggested that when six distributive and equivalent phosphorylations are required, Sic1 destruction is initially very slow when the first five sites are getting phosphorylated, then after a lag period, degradation dramatically speeds up. In another word, a temporal threshold is created for Sic1 destruction from the onset of Cln-CDK activation. Alternatively, if the degradation of Sic1 be governed by a single phosphorylation, there would have been a gradual decrease of Sic1 amount without time delay. The modeling framework presented by Deshaies and Ferrell in this review, albeit primitive, represents a very intriguing idea aiming to make the key connection between Sic1's observed phosphorylation threshold number to its ultimate function of regulating the G1/S transition. However, there remained several caveats. First, six phosphorylations were considered, while Sic1 has a total of nine sites. Do the remaining sites play any role? Second, it can be anticipated that exactly how the kinase is activated, i.e. the temporal profile of the kinase signal, affects the degradation of the substrate protein, and this aspect was not discussed. Finally, it was not clear what determined quantitatively the temporal threshold and speed of degradation. In this work, we will attempt to address the above issues, by carrying out systematic and detailed mathematical modeling to examine how multisite phosphorylation might lead to switch-like protein degradation.

Switch-like behaviors have been studied extensively in the steady state domain, where the response of a biological system (e.g. the amount of oxygen bound by the hemoglobin protein in response to the change of oxygen concentration) exhibits the very intriguing property of buffering fluctuations in the stimulus below a threshold and amplifying drastically the change of stimulus above the threshold. This type of switch-like response in the steady state domain has been termed “ultrasensitivity” in the literature and a number of mechanisms have been proposed to account for its sources, including ligand cooperativity [27], multi-step effect [28]–[30], enzyme saturation (i.e. zero-order ultrasensitivity) [31], [32], positive feedback [33]–[36], multi-level cascade [37]–[39], multisite phosphorylation [9], [32], [40]–[44], and substrate competition [45]. These studies have generated important insights concerning steady-state responses, which often correspond to *in vitro* experimental assays, on how switch-like behaviors arise. Nevertheless, what is crucial for many systems, particularly *in vivo* processes, is the transient stimulus-response curve such as the temporal profile of Sic1 during the G1/S transition, whereas switch-like responses in the temporal domain have been investigated very limitedly. It should be noted that the cell cycle signaling network in yeast has been examined very extensively through mathematical modeling [46]–[49]; however, degradation of macromolecules such as Sic1 has been modeled with a single phosphorylation reaction without taking into account the multiple phosphorylation steps.

In a previous work, we showed that multisite phosphorylation is a potential source of switch-like steady-state responses; most importantly, a large number of total sites combined with an intermediate threshold number of sites for changing substrate functionality account for the switch-like behavior (manuscript in revision). Here, extending our previous studies, we investigate switch-like responses in the time domain when protein stability depends on the degree of phosphorylation. We will present a model to analyze phosphorylation-triggered elimination of the substrate protein in response to the rise of kinase activity. We will show quantitatively how different parameters affect the protein elimination dynamics when degradation occurs above a threshold number of phosphorylations. In particular, we will explore how the extent to which the degradation dynamics is switch-like is affected by the type of kinase stimuli, the number of phosphorylation sites, the order of phosphorylation reactions, and kinetic parameters. We have developed the model mainly based on the Sic1 system to reveal the role of its existing multiple sites in regulating the protein's switch-like destruction during the G1/S transition. However, multisite phosphorylation is potentially a widespread source of switch-like protein degradation and the design principles revealed by our model might be applicable to many other multisite regulatory proteins.

## Results

### A general model describing the dynamics of multisite-phosphorylation-triggered protein degradation

**Figure 1:**
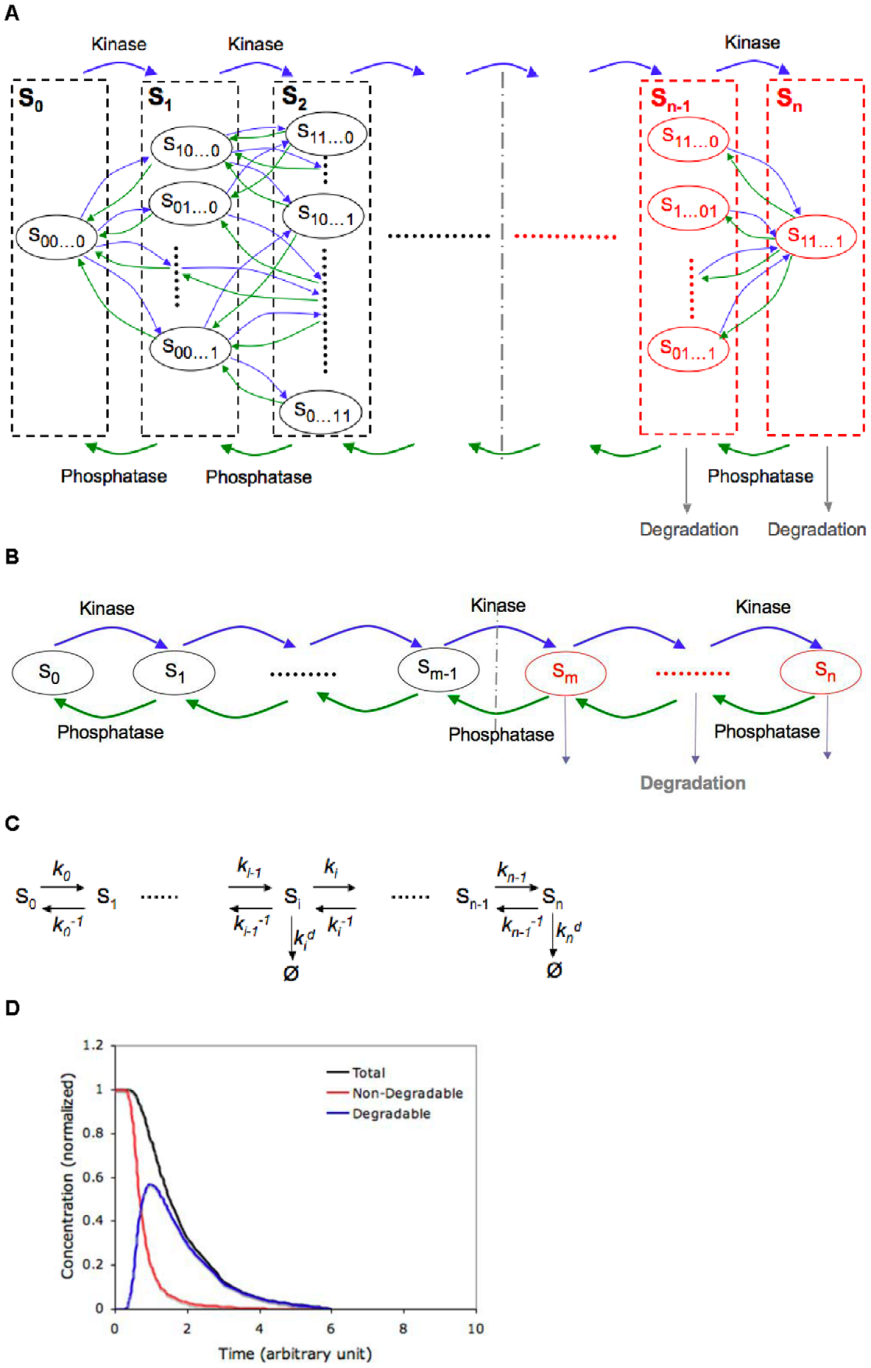
Phosphorylation-triggered degradation of a multisite protein. (A) A single kinase and a single phosphatase act on an 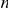-site protein in a random order. Each oval represents a distinctly phosphorylated state of the substrate protein S and the subscript indicates whether or not each site is phosphorylated (e.g. 10100 means that the 1st and 3rd sites out of fives sites are phosphorylated). The protein becomes degradable (highlighted in red) if 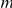 or more sites are phosphorylated. (B) The special case of a fully sequential system. The superscript of S indicates how many sites are phosphorylated. (C) Kinetic parameters for sequential phosphorylation and dephosphorylation: 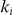, 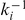 and 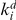 stand for kinetic rate constants of phosphorylation, dephosphorylation and degradation, respectively. 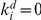 for 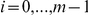 and 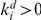 for 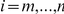. (D) The output of the model is shown for a random system with the following parameters: 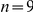, 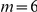, 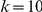, 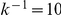, 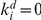 for 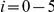 and 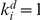 for 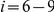, 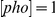, 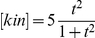.

Figure 1A illustrates the phosphorylation-dephosphorylation-degradation reaction network of a hypothetical protein with 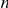 phosphorylation sites. We consider a single kinase and a single phosphatase acting on the substrate. We assume that each phosphorylation or dephosphorylation reaction involves an independent collision between the enzyme and the substrate; i.e., reactions proceed distributively. The number of distinguishable phosphorylated species depends on the order of the phosphorylation and dephosphorylation reactions. Due to the lack of data in multi-step phosphorylation/dephosphorylation kinetics, currently, it is not clear whether there are specific major patterns across different systems. Therefore, in this paper, we consider the most general case in which the reactions happen in a random manner. In another word, any unphosphorylated (or phosphorylated) site can be phosphorylated (or dephosphorylated) at any time regardless of the states of other sites. Existing data on half-lives of Sic1 mutants suggested that phosphorylation on this protein's most sites (eight out of nine) might be largely random. We term such a system a random one, in which there are 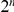 differently phosphorylated species and there exist 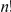 distinct pathways to move from the completely unphosphorylated state to the fully phosphorylated one. An important special case arises when the kinase/phosphatase chooses a specific site with 100% bias over the other sites at any time. In this case, the phosphorylation/dephosphorylation reactions proceed in an entirely ordered manner, as shown in Fig. 1B. There are evidences that certain proteins undergo phosphorylation in such sequential manners. For instance, 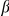-catenin, a cadherin associated protein in *Mammalia*, is phosphorylated by kinase Gsk3 on three sites sequentially during the G2/M-G1 transition [50]. Such systems are termed sequential here, which simplifies into 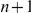 differently phosphorylated species and a single straight-line pathway from the unphosphorylated state to the fully phosphorylated one.

During proteolysis, once a substrate protein is phosphorylated properly, it is then ubiquitinated by a constitutively active SCF ubiquitin ligase [6] and thereafter degraded by the proteasome. In this work, for simplicity we modeled the degradation as a single elimination reaction. As mentioned previously, the protein Sic1 undergoes ubiquitination and proteolysis if it is phosphorylated on at least six sites [9]. In our model, we introduced a second parameter, 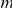, and generalized that once 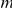 or more sites are phosphorylated, the substrate protein is recognized by the SCF complex and goes through degradation, as illustrated in Figure 1A&B.

Our interest is to investigate the temporal change of the substrate protein as the kinase level increases converting the substrate to degradable forms and causing it to be eliminated from the system. An ordinary differential equation (ODE) based approach was employed to examine this dynamic process. According to the Michaelis-Menten formalism for enzymatic reactions, the substrate reacts with the kinase to form a substrate-kinase complex that can in turn dissociate to form either the enzyme and substrate, or the enzyme and a product which has one more site phosphorylated. Similarly, the phosphorylated protein and the phosphatase react to form the substrate-phosphatase complex, which dissociates to form either the phosphatase and phosphorylated protein or the phosphatase and a product that has one less site phosphorylated. A complete mechanistic model with these elementary steps would consist of 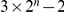 ODEs for all the substrate related species in the random system described above. It is possible, fortunately, to make substantial simplifications when i) the concentrations of both enzymes are much lower than the initial concentration of the substrate protein, or ii) the enzyme-substrate association and dissociation rate constants are much larger than the catalysis rate constants (see details in Note S1). The first condition holds readily for *in vitro* experiments. For *in vivo* systems, we do not know to what extent these two conditions would cover all circumstances, because of scarce kinetic information of multi-step phosphorylation/dephosphorylation reactions. Nevertheless, considering what is generally believed with regard to the rate-limiting step in enzymatic reactions, we believe that either of these conditions can be satisfied for a wide range of systems. Hence the model presented here potentially represents a phosphorylation-triggered degradation process that captures common features of many multisite proteins. Under the aforementioned two conditions, the enzyme-substrate complexes evolve in a much faster time scale compared with the free substrates and can be assumed to operate at quasi steady states. Consequently, they can be neglected in the model and the governing ODEs reduce to simplified forms as described below.

With the assumption that ATP is abundant, the concentration of each specific substrate state with a unique combination of phosphorylated and nonphosphorylated sites (termed phospho-state in this work) depends on the concentration of other phospho-states that are upstream or downstream in the phosphorylation-dephosphorylation network, the concentration of the kinase and phosphatase, as well as the kinetic parameters. For easiness of understanding, we illustrate first the equations for a sequential system which involves 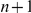 phospho-states in a straight-line pathway (see Figure 1B for reaction scheme and Figure 1C for kinetic parameter notations).
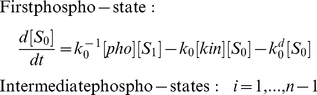
(1a)

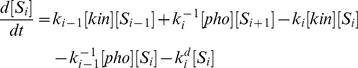
(1b)

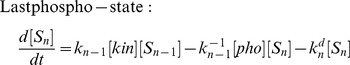
(1c)where 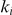, 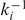 and 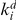 stand for kinetic rate constants of phosphorylation, dephosphorylation and degradation, respectively. 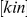, 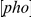 and 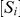 represent the concentration of the kinase, phosphatase and substrate state with 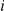 phosphorylated sites, respectively. It should be noted that first-order kinetics is used to describe the aggregated degradation reaction; 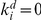 for 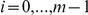 and 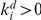 for 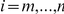. In this work, we further assume that 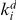 is the same for all phospho-states with 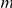 or more sites phosphorylated.

For a random system, there are 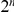 dependent variables representing concentrations of all the possible phospho-states. Each of them is determined by upstream/downstream reactions that produce/consume it, which can be mathematically described in a similar manner as in Eq. (1b) for the sequential system:
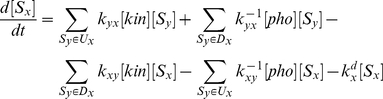
(2)where 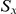 is any phospho-state in the phosphorylation-dephosphorylation-degradation network shown in Figure 1A; 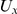 and 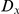 represent all the phospho-states upstream and downstream of 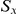, respectively. For example, for 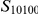, the set of upstream phospho-states with one less site phosphorylated 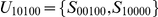 and the set of downstream phospho-states with one more site phosphorylated 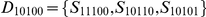. 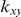 denotes the rate constant of the phosphorylation reaction converting 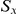 to 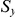; while 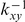 is that for the dephosphorylation reaction converting 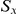 to 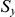. 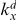 represent the degradation rate constant for 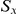. Despite exhibiting a seemingly more complicated form, Eq. (2) is structurally identical to Eq. (1b) and describes five types of reaction determining the change of each phospho-state: the first two positive terms represent increases due to phosphorylation of upstream phospho-state(s) and dephosphorylation of downstream phospho-state(s); the next two negative terms represent decreases due to phosphorylation to downstream phospho-state(s) and dephosphorylation to upstream phospho-state(s); the last negative term depicts degradation.

To simulate phosphorylation-triggered proteolysis, we assume that a basal activity of the phosphatase is always present in the system and the substrate exists in the unphosphorylated state initially. The kinase is then introduced into the system and increases gradually (detailed discussions will follow in the next section), converting the substrate to more and more phosphorylated states. As the substrate becomes sufficiently phosphorylated (i.e. on at least 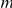 of the 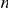 sites), it is eliminated through the degradation reaction, which causes the overall amount of the substrate to decreases continuously as proteolysis occurs. Figure 1D illustrates a typical simulation result, where the total, degradable and non-degradable amounts of the substrate are depicted. Inspired by a previous conjecture that multisite phosphorylation enables temporal thresholds [26], we hypothesize that precise timing of concentration decreases is crucial for certain proteins such as those regulating cell cycle transitions. Accordingly, our main interest in this work centers on the shape of the response curve representing concentration changes of the substrate. By applying standard non-dimensionalization techniques (see Note S2), we can reduce all the parameters to two sets, specifically 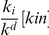 and 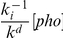, where 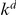 is the rate constant for the degradable states. Subsequently, we focus on examining the normalized concentrations of the substrate versus dimensionless time (i.e. in some properly selected time scale).

By “precise timing of concentration decrease”, we mean two features: i) starting from the time the kinase concentration increases, there is no appreciable change in the substrate concentration until a critical moment (i.e. temporal thresholding), and ii) the substrate concentration decreases immediately after passing the temporal threshold. Such a *switch-like* degradation process essentially enables the substrate protein to exist at two distinct levels separated in time, which might be a fundamental component in the mechanism of discrete and often irreversible cellular decisions. Graphically, these response curves exhibit the characteristic reverse-sigmoid shape, as illustrated by the red curve for the non-degradable substrate in Figure 1D. To what extent the degradation is switch-like can be quantified by the steepness of the response curve. Borrowing the basic idea of response coefficients used in measuring ultrasensitivity in steady state responses [31], here we define a response coefficient in the time domain to characterize the steepness of the response curve for phosphorylation-triggered protein degradation:
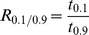
(3)where 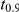 and 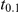 represent the times at which the substrate concentration decreases to 90% and 10% of the initial amount, since the kinase level starts increasing. The closer this value is to one, the steeper the reverse-sigmoid response curve appears and the more switch-like the degradation is. We can further extend this index to examine related local properties of the response curve. More specifically, the following two partial response coefficients can be used to indicate how steep the decrease of the substrate is during the first and second halves of the degradation process, respectively.
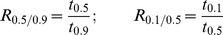
(4)where a middle time point 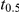, the time at which the substrate reaches exactly half of the original concentration, is incorporated. It is worth noting that the overall response coefficient, 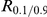, is the product of these two partial response coefficients. It means that the overall process is switch-like if and only if the response curve is highly nonlinear both in the early and in the late phases.

### The stimulus profile greatly influences the response curve

**Figure 2:**
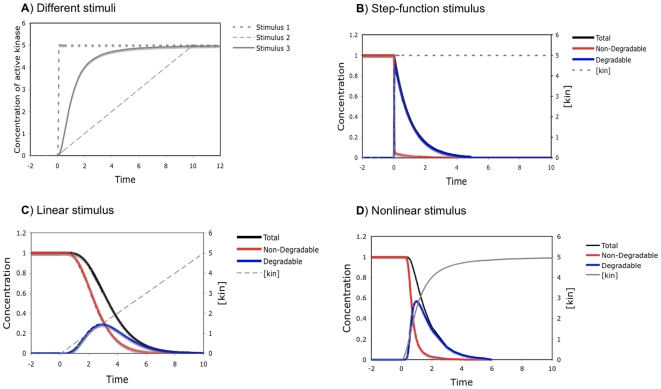
Responses of a nine-site protein to three types of kinase stimuli. (A) The concentration of active kinase increases from zero to a maximum value in three different manners: a step function (Stimulus 1, 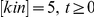); a linear function (Stimulus 2, 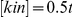); and a nonlinear function (Stimulus 3, e.g. 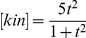). (B–D) Responses of a random system to the three stimuli with the following parameters: 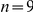, 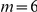, 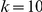, 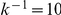, and 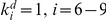, 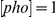.

The stimulus in our model is the increase of kinase level, but very little is known concerning exactly how a kinase rises in phosphorylation-triggered protein degradation. We started out by considering three types of stimuli that differ in how the level of active kinase changes from zero to the same maximum value within the same period of time: step-function increase; linear increase; and nonlinear increase. Figure 2 illustrates these three types of kinase stimuli and how a nine-site substrate gets eliminated in response to each of them. It is very clear that the kinase profile greatly affects when and how rapidly the substrate disappears. As shown in Figure 2B, when the kinase is sharply increased like a step function, the substrate level decreases immediately in an exponential manner, which could be very rapid. This type of stimuli is an ideal case and is likely to be valid only for *in vitro* experiments when the kinase is added instantaneously to a phosphorylation-dependent degradation system. Under *in vivo* conditions, the rise of the kinase can only occur gradually, either in a linear or nonlinear fashion. For nonlinear stimuli, we use a sigmoid function with the general formula of 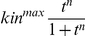, the sharpness of which can be tuned by parameter 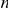. As exemplified in Figure 2C&D, now the substrate disappears at slower rates, compared with that in response to a step-function kinase increase, and we can observe temporal delays in the substrate decrease. Both the total and non-degradable amounts of the substrate follow a reverse-sigmoid curve, hence we can quantify to what extent the response is switch-like (i.e. combining temporal thresholding and rapid decrease after the threshold) using the response coefficients introduced above. Not surprisingly, the sharper the kinase profile is, the faster and more switch-like the degradation is. For example, a sharper (nonlinear) increase of the kinase in Figure 2D leads to steeper response curves of the total and non-degradable substrate, compared to the linear kinase increase in Figure 2C. Therefore, the exact response of a multisite protein in such a phosphorylation-triggered degradation process is determined by both the molecular properties of the substrate and the kinase stimulus. Our main focus, in this work, is to examine whether and how various properties of a multisite protein can enable it to behave like a molecular switch, converting graded inputs to discrete outputs. Consequently, for further investigation, we chose to continue with the sigmoid function for specifying the kinase stimulus, which exhibits flexible shapes and may capture well many temporal profiles in real systems. Furthermore, it can be shown that as sharper and sharper stimulus curves are specified (by increasing parameter 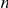), improvement of the response coefficient becomes less and less significant (see Note S3). Accordingly, we use the sigmoid stimulus with 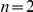 as a standard in most of our work. Within this context, we call a response curve representing the temporal profile of the substrate “switch-like” if its steepness is higher than that of the stimulus curve of the kinase, which can be determined quantitatively by comparison of the response coefficient and its counterpart for the stimulus curve.

**Figure 3:**
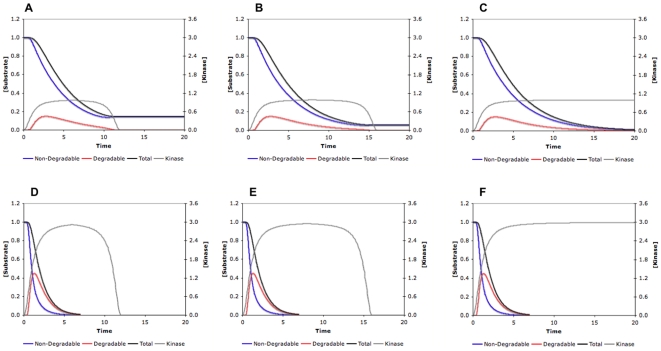
Effect of stimulus strength and duration. (A–C) A weak stimulus with three durations. (D–F) A strong stimulus with the same three durations. The shortest duration is 12 in (A) & (D): 
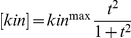
 for 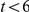 , 
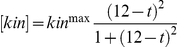
 for 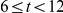, and 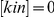 for 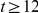. The intermediate duration is 16 in (B) & (E). Parameters: random system, 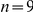, 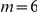, 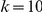, 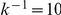, 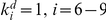, 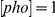. With this set of parameters, the minimum value of 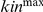 that leads to removal of at least 99% of the substrate during the rising phase of the kinase is 1.7.

Another relevant property of the stimulus is its duration. It is very likely that the kinase concentration will not be maintained permanently after it reaches the maximum. Instead, it will decrease gradually in a period of time because of degradation or other reasons. This is the case for many cyclin-dependent kinases, which rise and fall periodically during the cell cycle. For example, in the Sic1 system, the concentration of Cln2, which activates the Cdc28 kinase for Sic1 phosphorylation, increases gradually in the G1 phase, reaches its maximum before budding (beginning of the S phase), and then decreases [20], [51]. We investigated the effect of stimulus duration and our simulation revealed a tradeoff between stimulus duration and strength. Figure 3 compares the response curves resulted from a weak stimulus and a strong one with three durations. As shown in Figure 3A–C, when the stimulus strength, determined by the maximum kinase level, is low, the substrate decreases slowly and a substantial amount still remains when the kinase level reaches the maximum. In this case, the duration of the kinase is important and affects to what extent the substrate can be eliminated. For example, the duration of the kinase in Figure 3A is not sufficient to remove 90% of the substrate. In turn, if it is required (e.g. for achieving an associated cellular regulation) to reduce the substrate to at most 10% of its initial level, the kinase would need to be maintained for a longer period of time such as the one in Figure 3B. However, when the stimulus strength is above certain critical level, the substrate has been largely eliminated by the time the kinase reaches the maximum and hence whether the kinase is maintained afterwards does not matter, as demonstrated in Figure 3D–F. This apparent tradeoff between kinase strength and duration is part of the challenge in resource allocation a cell constantly faces. We hypothesize that for certain protein degradation processes associated with critical regulations such as those in cell cycle progression, it is more desirable for the cell to generate a strong while short stimulus to achieve fast and robust degradation. Accordingly, for the rest of this paper, we will consider stimulus strength above the critical level, sufficient to eliminate the majority of the substrate during the rise of the kinase.

### Effects of 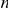, 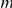, and order of phosphorylation/dephosphorylation

With well-defined kinase stimulus, we now turn to the main puzzle of whether a multisite protein is switch-like in its degradation, most importantly, why multiple sites may help to achieve this property. In addition, we want to address the question of how the extent to which the degradation dynamics is switch-like is determined quantitatively.

As described above, it is assumed that a protein with 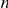 phosphorylation sites is degradable when it is phosphorylated on at least 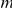 sites. As a case study, different values of 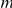 have been examined for 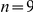 to model the Sic1 degradation. A question that one may raise here is which kind of Sic1 matters for the cell, the non-degradable fraction or the total protein (including the degradable and non-degradable fractions). The answer depends on the biochemistry of how Sic1 inhibits cyclins required for DNA replication and how it gets ubiquitinated. If Sic1 releases Clb5/6 as soon as it is ubiquitinated by the SCF complex, then only the non-degradable fraction is important as the CDK inhibitor. Otherwise, if Clb5/6 is released from Sic1 only when Sic1 is destructed by the proteosome, the total amount of Sic1 should be considered. Unfortunately, it is not known which case of the above is true. Therefore, we examined the dynamic profiles of the non-degradable fraction and also the total amount of the protein during the degradation process for three different scenarios: 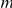 = 1, 5 and 9.

**Figure 4:**
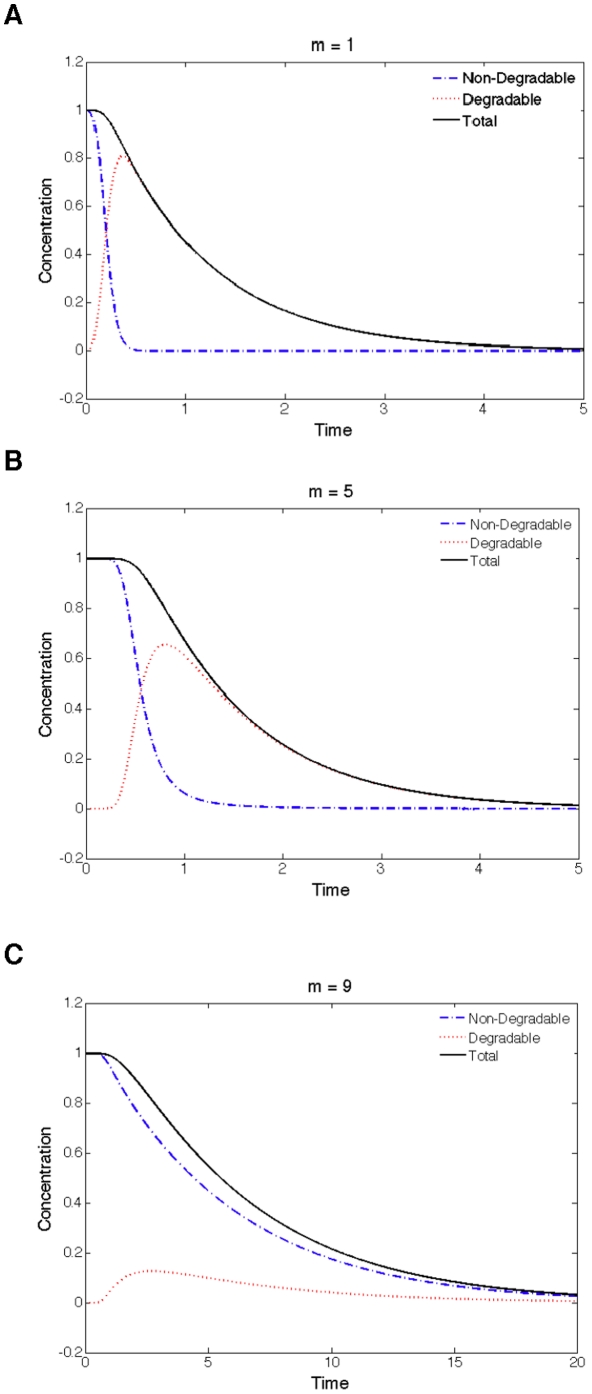
Temporal responses of a nine-site random system to different values of *m*. The most switch-like response takes places when 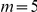. (A) 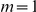, 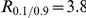. (B) 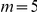, 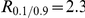. (C) 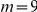, 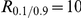. System parameters: 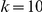, 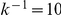, 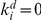 for 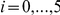 and 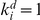 for 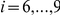, 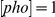, 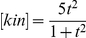.

As shown in Figure 4, the temporal profile of the total protein does not change much when 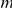 is varied. However, as far as the non-degaradble fraction is concerned, its temporal profiles differ significantly for different 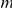 values. Specifically, the response coefficient 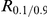 for 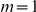, 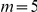, and 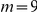 is 3.8, 2.3, and 10, respectively. Clearly, 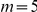 leads to the smallest response coefficient and thus the most switch-like degradation dynamics. In this scenario, the profile of the non-degaradble fraction features both an observable temporal threshold and rapid decrease after passing the threshold. In contrast, the profile for 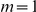 shows no temporal threshold; while for 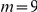, even though the profile also exhibits a temporal threshold, it suffers a long tail during the later period of the response. The highly nonlinear response of the system when 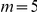 can be attributed to the two distinct sub-chains of phospho-states created in this case. The non-degradable sub-chain can buffer the kinase signal in the early phase, thus creating the temporal threshold. While during the later phase of the response after passing the temporal threshold, the degradable sub-chain draws the substrate protein effectively, even before the substrate undergoes final destruction, and subsequently reduces the non-degradable pool very rapidly.

We can quantify the nonlinearity of the kinase stimulus with a stimulus coefficient similar to the response coefficient: 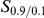, the ratio between the time at which the kinase reaches 90% of the maximum and the time at which it reaches 10%. The stimulus we have used corresponds to a 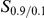 of 9 and in the case of 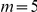, this gradually increasing stimulus causes a switch-like decrease of the non-degradable forms of the protein with an 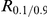 of 2.3. In another word, a single macromolecule can increase the nonlinearity of the system by four times. These results demonstrate the potential capability of multisite proteins in creating biological switches.

The above results may explain why in *S. cerevisiae* phosphorylation on at least six out of nine sites is required for Sic1 degradation. According to our model, this design would enable the Sic1 protein to respond to the kinase signal in a highly switch-like manner: the non-degradable fraction of this CDK inhibitor does not change appreciably before a temporal threshold even though kinase has risen, then once the temporal threshold is passed, the non-degradable fraction decreases very rapidly. We hypothesize that this switch-like degradation is crucial for Sic1's regulatory function during the G1/S transition of the cell cycle. Otherwise, if Sic1 could bind to Cdc4 for ubiquitination and subsequent degradation, either as soon as it was phosphorylated on a single site (i.e. 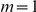), or only when it was fully phosphorylated (i.e. 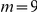), elimination of Sic1 would not have been highly switch-like and the G1/S transition could not have occurred in an yes/no manner. This hypothesis will require future experimental validations and specific assays can be designed to test various components of this theory. For instance, it will be interesting to examine experimentally whether or not Sic1 in a complex with Cdc4 (i.e. the degradable form of Sic1) can still inhibit Clb5/6.

**Figure 5:**
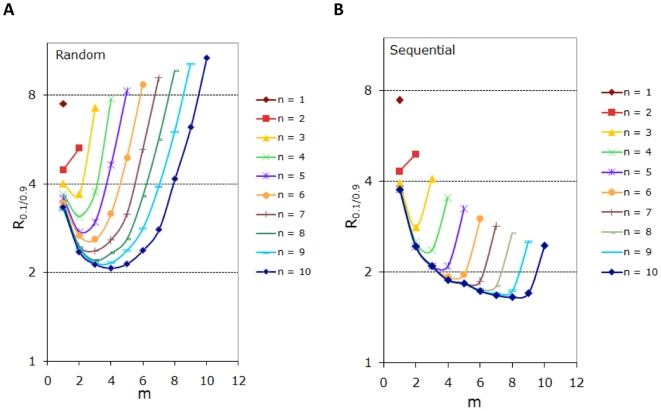
Effects of the number of phosphorylation sites (*n*) and the threshold for degradation (*m*). The response coefficient of the non-degradable fraction for different values of *n* and *m* (*m* varies from 1 to *n*) are shown for random (A) and sequential (B) processes. System parameters: 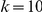, 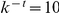,  for 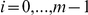 and 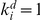 for 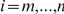, 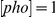, 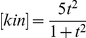.

In the rest of this study, we will build on the above hypothesis, i.e. we will focus on exactly how the non-degradable fraction of the substrate protein decreases in response to a rise of the kinase. Here, we first examine the effects of the total number of sites, 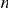, and the threshold number for degradation, 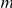. Our simulation results are summarized in Figure 5A for random phosphorylation/dephosphorylation and in Figure 5B for the sequential process. Several conclusions can be drawn based on these results. First, for both random and sequential processes, in general, the mores sites a protein has, the more switch-like it can be in its degradation dynamics. The response coefficient 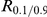 for 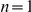 is about 7.5, which is not much smaller than the stimulus coefficient of 9. Therefore a single-site protein does not generate an output that is significantly sharper than the input. As the total number of sites, 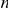, increases, the achievable response coefficient 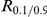 decreases, i.e. the protein can degrade in a more and more switch-like manner. Second, the threshold site number for degradation, 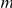, plays an important role in determining the exact extent to which the response is switch-like. For random processes, the smallest response coefficient 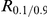 (i.e. the most switch-like response) is achieved when 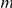 is close to and often slightly smaller than half of 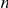 (e.g. 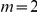 for 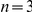, 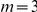 for 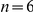, and 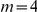 for 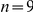). In contrast, for sequential processes, an 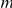 value only slightly smaller than 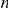 delivers the most switch-like response (e.g. 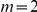 for 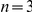, 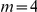 for 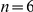, and 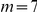 for 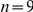). Finally, the response of a sequential system is generally more switch-like than that of a random one for given values of 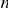 and 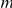.

### Effects of kinetic parameters

Next, we will examine another important factor that affect the degradation dynamics - the kinetic parameters. In the model presented above, there are three groups of kinetic parameters, associated with phosphorylation, dephosphorylation and degradation reactions respectively. We have focused on two specific effects and conducted sensitivity analysis.

#### Change of phosphorylation and dephosphorylation kinetics along the chain

Phosphorylation reactions have been studied extensively, nevertheless, kinetic data of multi-step phosphorylation remain scarce. One exception is the transcription factor Pho4 in the phosphate-responsive signaling pathway in the budding yeast. Pho4 contains five phosphorylable sites for kinase Pho80–Pho85. Kinetic parameters of Pho4 phosphorylation by Pho80–Pho85 have been measured through integrating experimental data and computational modeling by Jeffery and colleagues [52]. It was found that the phosphorylation rate constants for all of the five phosphorylation steps are of the same order of magnitude. Based on this data, we assumed in the above analysis that the rate constants of all the phosphorylation reactions are the same. In terms of dephosphorylation, even less data is presently available on its multi-step kinetics.

**Figure 6:**
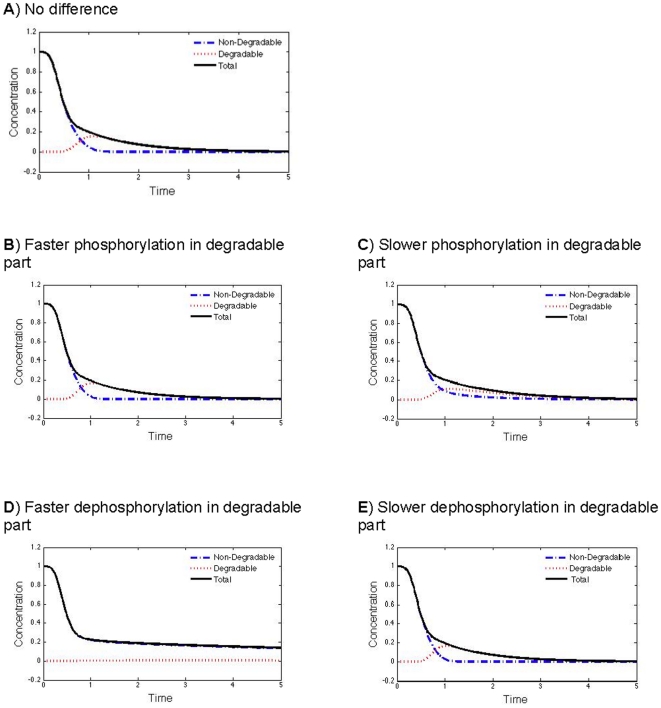
Effects of changes of phosphorylation and dephosphorylation rate constants along the chain. (A) No difference between degradable and non-degradable fraction in terms of kinetic rates of phosphorylation and dephosphorylation reactions: 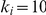 and 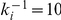 for 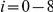. (B) The kinase phosphorylates the degradable fraction faster than the non-degradable fraction: 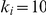 for 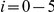 and 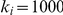 for 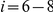. (C) The kinase phosphorylates the degradable fraction slower than the non-degradable fraction: 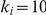 for 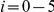 and 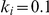 for 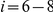. (D) The phosphatase dephosphorylates the degradable fraction faster than the non-degradable fraction: 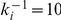 for 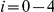 and 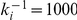 for 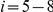. (E) The phosphatase dephosphorylates the degradable fraction slower than the non-degradable fraction: 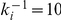 for 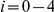 and 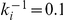 for 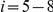. The outputs are shown for a sequential system with the following parameters : 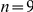, 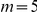, 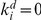 for 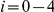 and 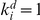 for 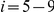, 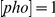, 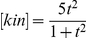.

Given that only very limited data is available for the multi-step kinetics of phosphorylation/dephophorylation, we would explore the possible scenario that earlier phosphorylation/dephosphorylation steps may suppress or enhance subsequent phosphorylation/dephosphorylation of remaining sites, especially if the exact phospho-state affects the substrate's configuration (e.g. enabling or disabling the binding of a downstream protein in the degradation pathway), and thus the kinetic parameters may change along the phosphorylation/dephosphorylation chain. Here, we consider the case where the phosphorylation/dephosphorylation rate constants might increase or decrease after passing the threshold for degradation. As the comparison of five cases shows in Figure 6, the degradation dynamics of the substrate, i.e. the temporal profile of the non-degradable fraction of the protein, does not change much if the net phosphorylation rate (i.e. phosphorylation against dephosphorylation) is faster than that of the degradation reaction (see Figure 6A,B,E). On the other hand, if the phosphorylation steps for the degradable part is too slow compared to the degradation reaction, due to either slower phosphorylation (Figure 6C) or faster dephosphorylation (Figure 6D), the elimination of the non-degradable fraction of the protein becomes less switch-like, as indicated by the less rapid drop after the initial delay.

#### Rates of phosphorylation/dephosphorylation vs. degradation

**Figure 7:**
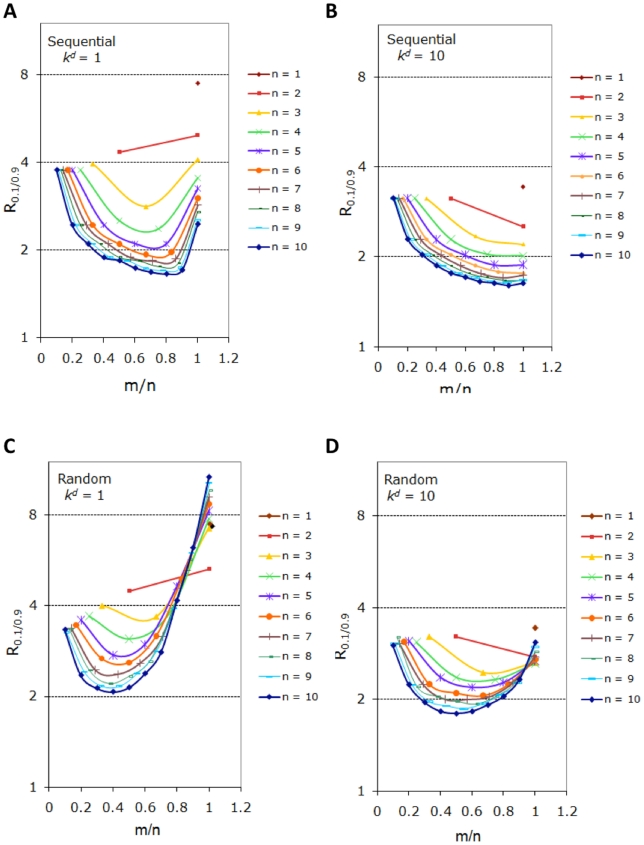
Rates of phosphorylation/dephosphorylation vs. degradation. Systems with slow and fast degradation rate constants are compared in terms of sharpness of the temporal profile of the non-degradable fraction for sequential (A–B) and random (C–D) processes. The response coefficients are shown for the following parameters: 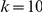, 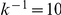, 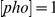, 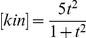.

As described above, our model consolidates ubiquitination and destruction by proteasome into one single degradation reaction. Relatively little is known about the kinetics of ubiquitination in general. Fortunately, a recent study has revealed the kinetics of polyubiquitylation of Sic1 [53], which is slower than the multi-step phosphorylation kinetics of Pho4. In light of this evidence, we have assumed that the degradation is slower than phosphoryltation/dephosphorylation for most of the analysis presented above. Nevertheless, given the limited data currently available and the diversity that might exist in related molecular events, here we explore how the relative rates of phosphorylation/dephosphorylation vs. degradation affect the degradation dynamics. Specifically, we compare the response coefficient 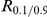 of two cases: in one, the degradation is slower than phosphorylation/dephosphorylation (Figure 7A&C, which are alternative plots of Figure 5); in the other, the degradation is comparable to phosphorylation/dephosphorylation (Figure 7B&D). It is found that for sequential processes, if the degradation is as fast as phosphorylation/dephosphorylation, the elimination of the non-degradable substrate becomes more switch-like, as indicated by the smaller 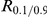 in Figure 7B compared to that in Figure 7A. However, the smallest 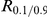 is now achieved when 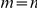, i.e. the advantage of having a large number of sites (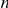) and an intermediate threshold (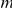) in creating a switch-like response vanishes. For random processes, faster degradation also leads to smaller 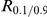, while the most switch-like response still occurs when 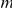 is close to half of 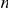 (see Figure 7C&D). It remains to be seen whether nature utilizes all these different regimes in the design of protein degradation switches.

#### Sensitivity analysis

Our model of multisite phosphorylation triggered protein degradation is subject to many sources of uncertainty, including lack/inaccuracy of data and biological fluctuations. To investigate how variations in kinetic parameters affect the model output (i.e. the response coefficient of non-degradable substrate) we conducted sensitivity analysis. As a demonstration, here we focus on a sequential system with 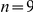 and 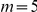. There are a total of nine rate constants for phosphorylation, nine rate constants for dephosphorylation, and five non-zero rate constants for degradation.

First, we examine how perturbation of each parameter affects the model output. We choose to change the parameter within 20% of its nominal value which correspond to the base scenario discussed above (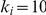 and 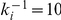 for 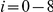, 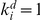 for 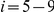). It was found that such perturbations result in very insignificant changes of the response coefficient for the non-degradable substrates (less than 2%). This analysis also shows that each parameter affects the response coefficient in a monotonic manner (see examples in Figure S1). Specifically, increasing a phosphorylation rate constant decreases the response coefficient; increasing a dephosphorylation constant increases it; while the change of a degradation rate constant has no appreciable effect.

**Figure 8:**
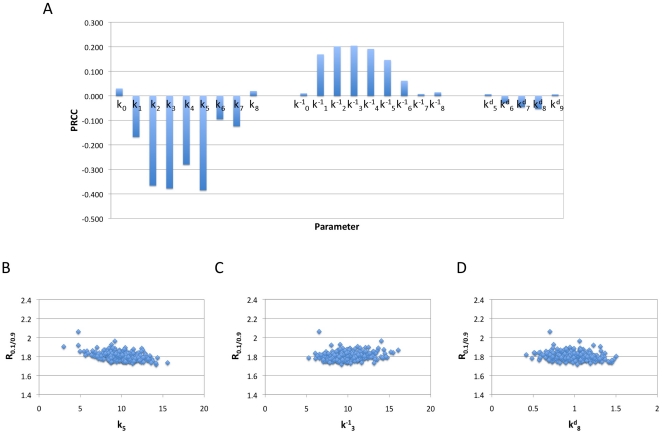
Sensitivity analysis for phosphorylation, dephosphorylation and degradation rate constants. Partial rank correlation coefficients calculated for all nine phosphorylation rate constants, nine dephosphorylation rate constants and five non-zero degradation rate constants (A). Scatter plots of the non-degradable substrate's response coefficient for 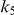 (B), 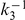 (C) and 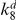 (D). Results are based on 1000 simulations for a sequential system with 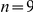, 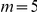, 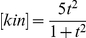. The parameters are picked randomly from normally distributed space where the means correspond to the base scenario (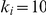 and 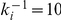 for 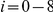, 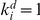 for 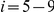) and the standard deviation is set to be 20% of the mean.

Then, we perturb all the parameters simultaneously while independently and examine their effect on the model output. We randomly choose the parameter values based on a normal distribution where the mean correspond to the base scenario discussed above and the standard deviation is set to be 20% of the mean. This sampling was conducted 1000 times. The mean of the model output (i.e. the response coefficient of non-degradable substrate) is 1.81 and the standard deviation is 0.04, about 2.2% of the mean. We were also interested in how each parameter correlates with the model output in this context and calculated the partial rank correlation coefficient (PRCC). PRCC is a robust measure of sensitivity for nonlinear but monotonic relationships between a certain input (a parameter in our context) and the output as long as little or no correlation exists between the inputs [54]. As shown in Figure 8A, the degradation rate constants show negligible correlation with the sharpness of the temporal profile of the non-degradable substrate; while the phosphorylation and dephosphorylation parameters show significant correlations. Increasing the phosphorylation rate constants shifts the system toward the degradable fraction, which leads to faster depletion of the non-degradable substrate and thereby smaller response coefficient. Decreasing the dephosphorylation kinetic parameters has the same effect, which explains the positive correlation coefficient. Figure 8B–D illustrate the scatter plots for three parameters of 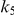, 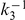, and 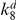, which show the largest correlation coefficient in each set of parameters.

The above analysis assumes simultaneous and independent perturbation of all the parameters which accounts for certain types of uncertainty such as stochastic fluctuations of kinetics. There might also exist uncertainty associated with the enzymes that affects a whole set of parameters. Thus, in another analysis, we considered a total of three parameters in our model (one for all the phosphorylation reactions, one for all the dephosphorylation reactions, and one for all the degradation reactions) and then perturbed them in a similar manner. Results from 1000 samplings and simulations led to a mean response coefficient of 1.81 and a standard deviation of 0.09 (i.e. 5% of the mean).

These results clearly show that the switch-like behavior of a multisite protein's degradation is very robust against random fluctuations of kinetic parameters.

### Effect of site preference

Finally, we will consider the effect of site preference on the degradation dynamics. In the completely random system described above, no site is preferred over other sites in term of the affinity for the kinase. However, for real proteins, due to effect of neighborhood amino acids, usually some sites are more likely to associate with the kinase and become phosphorylated. Consequently some pathways in the phosphorylation/dephosphorylation network are more dominant than others. A well-studied example is the extracellular-signal-regulated protein kinase ERK of the MAPK cascade. ERK contains two phosphorylable sites, Thr188 and Tyr190, for MEK kinase. The estimated kinetic parameters indicate that phosphorylation of Tyr is much faster than phosphorylation of Thr [55], with the corresponding rate constants differing by one order of magnitude. In the Sic1 system, Thr45 is preferred for phosphorylation by Cln2-Cdc28 over other sites [9]. In the Pho4 system, SP6 is preferred over other sites [52]. This property of site preference can be readily incorporated in our model by assigning a larger value of the phosphorylation rate constant for phosphorylation of the preferred site.

**Figure 9:**
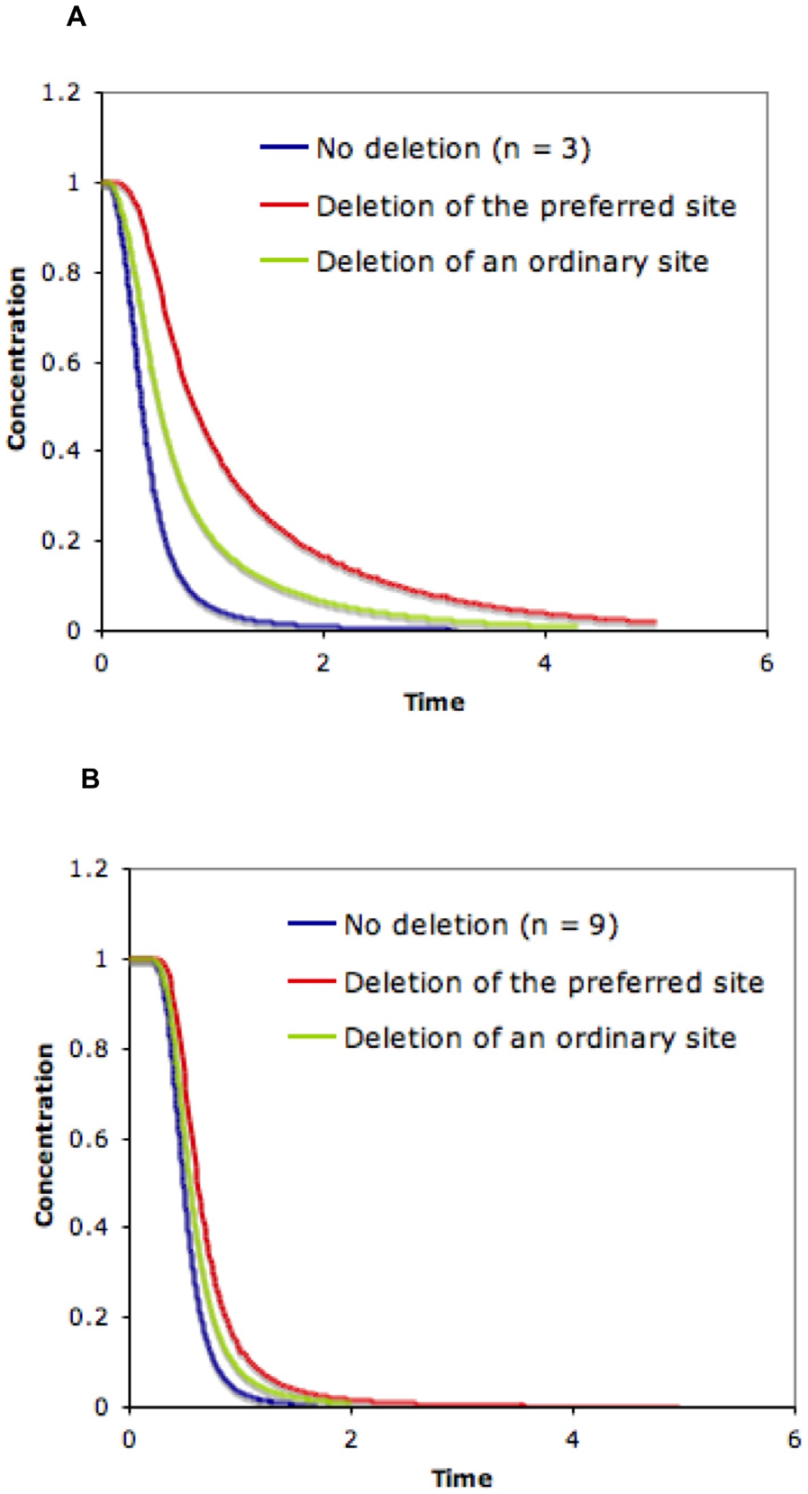
Effect of site preference. One site is preferred over other sites for the kinase. The deletion of this site is compared with deletion of an ordinary site for three-site (A) and nine-site (B) proteins in terms of sharpness of the temporal profile of the non-degradable fraction. Results are shown for a random system with the following parameters : 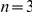, 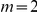 for A and 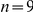, 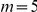 for B, 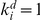, 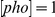, 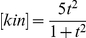. The phosphorylation rate constant for the preferred site is 100 times larger than that for the other sites.

When site preference exists, as expected, our model predicts that deletion of a preferred site has a stronger effect in both increasing the half-life of the protein and reducing the sharpness of the response curve, compared to deletion of ordinary sites (see Figure 9). However, this effect becomes less significant as the number of phosphorylation sites increases. In another word, the role of a given site, even a preferred one, in the response of the system to the kinase becomes less significant when the number of total sites increases. This result suggests that another advantage of having a larger number of sites might be enhanced robustness. If the substrate protein contains many sites, removal of one site (e.g. due to adverse mutations) is less likely to cause its function to break down, i.e. the system can tolerate better perturbations.

## Discussion

In this paper, we have analyzed systematically a general model for multisite-phosphorylation-triggered protein degradation processes. The model has been developed largely based on what were revealed experimentally for Sic1, a nine-site protein in *S. cerevisiae*, during its degradation at the G1/S transition of the cell cycle. Most importantly, the protein becomes degradable upon phosphorylation on a critical number of sites [9]. Inspired by subsequent theoretical conjectures concerning the role of multisite phosphorylation in regulating cellular dynamics [26], we set out to address the questions of whether and how multisite phosphorylations can cause a protein to respond to a gradually changing kinase signal and degrade in a highly switch-like manner. Here, we focus on switch-like transient responses, the characteristics of which include both temporal thresholding and rapid elimination beyond the threshold point. The temporal profile of the protein in such a process exhibits a reverse sigmoidal shape and our main interest is the steepness of the curve, which we quantify with the response coefficient 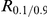, defined as the ratio between the time taken to decrease to 10% of the original protein concentration versus that taken to reach 90%. Our extensive simulation study showed that multisite phosphorylation is indeed capable of generating a degradation switch. In addition, we examined systematically how the extent to which the degradation is switch-like is determined by various features of the system, including the total number of sites, the threshold site number for degradation, the order of phosphorylation/dephosphorylation, the kinetic parameters, and site preference in phosphorylation.

Given the mostly unknown parameters involved in our model, it remains to be tested whether the exact predictions agree with actual degradation of multisite proteins such as Sic1. On the other hand, multisite phosphorylation has emerged as a recurring theme as researchers dissect the proteasome-dependent degradation pathway of various proteins in diverse species. Table 1 illustrates some of these examples we were able to compile readily by conducting a literature search on multisite phosphorylation and protein degradation. It is worth noting that many of these proteins with multiple phosphorylation sites involved in degradation play important roles in the regulation of the cell cycle, which is not surprising when one considers the extreme importance of exact timings of a cascade of events required for correct cell cycle progression. For instance, the regulation of Rum1, the functional homolog of Sic1 in *Schizosaccharomyces pombe*, might also involve up to eight phosphorylation sites [56]. As an example in higher organisms, RUNX1, a transcription factor associated with acute myeloblastic leukemia with maturation (M2 AML), degrades at the G2/M-G1 transition and the extent of phosphorylation on its eleven sites was suggested to play a role [57]. In addition to cell cycle control, precise protein degradation could also be critical for many other regulatory processes, such as eliminating cross-talk of mating and filamentous growth in *Saccharomyces cerevisiae* [58] and light signaling in *Arabidopsis thaliana* [59]. In light of this widespreadness of the involvement of multisite phosphorylation in regulating protein degradation, it appears plausible that the molecular mechanism described by our model might represent a common design principle utilized by nature for constructing protein degradation switches. Certain details of our model may require modifications or extensions, for example, the ability of the substrate protein to bind to ubiquitin ligase and thus undergo proteasome-dependent degradation could increase gradually instead of in a step-wise fashion. However, the overall conclusion that multisite phosphorylation provides a highly effective and flexible platform for switch-like protein degradation is likely to apply to a wide range of proteins across different eukaryotic species.

**Table 1:** Proteins regulated through phosphorylation dependent degradation.

Protein	Species	Role	Degradation Time	Kinase(s)	No. of Sites*	Ref.
Sic1	*S. cerevisiae*	Cyclin-dependent kinase inhibitor	G1/S	Cln/Cdc28	9	[9]
Cdc6	*S. cerevisiae*	Replication initiator	G1/S	Clb/Cdc28	4	[67]
Cln2	*S. cerevisiae*	G1 cyclin	G1/S	Cdc28	7	[68]
Rum1	*S. pombe*	Cyclin-dependent kinase inhibitor	G1/S	Cdc2	2 (or 8?)	[56]
Cdc18	*S. pombe*	Replication initiator	G1/S	Cdc2	6	[69]
p27	*Mammalia*	Cyclin-dependent kinase inhibitor	G1/S	Cdk2, Src	4	[10]–[12]
Cyclin E	*Mammalia*	G1 cyclin	G1/S	Cdk2, Gsk3	4	[70], [71]
Cdc25A	*Mammalia*	Phosphatase (dephosphorylation of CDKs)	S	Chk1/2, ?	8	[72]
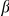 -catenin	*Mammalia*	Cadherin-associated protein	G2/M-G1	Gsk3, Cki 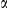	4	[50]
Runx1	*Mammalia*	Transcription factor (associated with M2 AML)	G2/M-G1	Cdk1	11	[57]
Emi1	*Mammalia*	Early mitotic inhibitor (inhibit APC)	M	Cdk1, Plk1	7	[73]
Gcn4	*S. cerevisiae*	Transcription activator	-	Srb10	5	[74]
		(biosynthesis of amino acids & purines)				
Tec1	*S. cerevisiae*	Filamentous growth regulator	In response to	Fus3	2	[58]
			mating pheromone			
HFR1	*A. thaliana*	Transcription factor in light regulation	In darkness	CKII	5	[59]
Bim	*Mammalia*	Apoptosis signaling	In response to	MAPK, JNK	4	[75]
			survival signals			

*Phosphorylation sites shown to be involved in the regulation of the stability of the corresponding protein.

The work presented here for phosphorylation-triggered degradation also has certain limitations. First of all, we used a simplified kinetic model, as explained above and in Note S1, and the underlying assumptions regarding enzyme/substrate concentrations or kinetic parameters may not be applicable to all biological systems. Second, we considered free enzymes in our model, while in experiments, it is usually the total enzyme concentration that can be measured. It remains to be explored, perhaps with an alternative model where enzyme-substrate complexes are included explicitly, how these two quantities relate to each other. Third, we examined two extreme cases of fully random and fully sequential phosphorylation. Real biological systems might be in between. Random processes may be favored in the evolution of biology as it might provide more intermediate forms of the phosphorylated protein and allow more flexibility for protein regulation. However, phosphorylation cannot proceed fully randomly and due to neighborhood effects some sites have higher affinity for the kinase or phosphatase. Finally, we assumed only one kinase is responsible for phosphorylation while it is known that for some substrates, multiple kinases might be involved. For the Sic1 protein, different kinases are involved in different processes or at different times. Cdc28 and Pho85 apparently phosphorylate Sic1 at several sites in late G1 in the budding yeast [60], [61]. However, Ck2 phosphorylates Sic1 at Ser201 shortly after Sic1 *de novo* synthesis [62]. Ime2 is another kinase which is necessary for timely destruction of Sic1 during sporulation [63]. In addition, Hog1, a stress-activated protein kinase phosphorylates a single residue which contributes to arresting at G1 phase in response to stresses such as high osmolarity [64]. Incorporation of multiple kinases in an extended model would potentially lead to further understanding of real protein degradation switches as well as signal integration.

The biggest obstacle in theoretical studies of protein phosphorylation and dephosphorylation is that biochemical parameters are not known for most biosystems; even for well-studied systems, parameters are obtained through *in vitro* experiments and under specific conditions which may not reflect biological realities. Another difficulty is the lack of qualitative knowledge on the phosphorylation process and degradation dynamics. Even the exact number of phosphorylation sites for a given protein is usually not provided in the literature. Of the proteins listed in Table 1, very few have been studied carefully regarding the quantitative connection between multisite phosphorylation and degradation dynamics.

A main challenge in quantitative understanding of phosphorylation and dephosphorylation processes is the identification and quantification of phosphorylated protein species. Mass spectrometry is becoming widely used as a fast, sensitive and high-throughput measurement method in the identification and quantification of protein phosphorylation. For instance, Olsen and colleagues detected and quantified phosphorylation of 6,600 sites on 2,244 proteins in response to a stimulus in mammalian cells through stable isotope labeling by amino acids in cell culture [65]. In most MS approaches, the substrate protein is digested into peptides and the degree of phosphorylation at one site is calculated based on the summed abundance of the peptides containing this site. For a protein such as Sic1 which has nine phosphorylation sites, a partially phosphorylated protein solution might contain 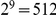 different phosphorylated states of the protein, whereas even in the ideal case where each peptide contains only one phosphorylation site, merely 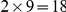 concentrations can be measured from MS. Although results of phosphopeptide concentrations can be utilized for certain characterizations such as determining site preference, they are not sufficient for estimating kinetic parameters. New approaches are needed to address this issue. For example, one might consider making use of a set of mutants, each with a subset of the phosphorylation sites, in kinetic assays and then integrating the data systematically.

In summary, in this paper, we investigated the capability of a single protein with multiple phosphorylation sites in converting a graded input to a switch-like output signal in the time domain. We would like to point out that besides phosphorylation, a dominant post-translational modification for regulating protein stability and activity, other types of modification such as ubiquitination, methylation and glycosilation have also been shown to be involved in tuning the stability, activity and translocation of macromolecules. The model presented here or its variations can potentially explain and predict the behavior of these multisite modification systems as well. Finally, as have been demonstrated by a synthetic single-molecule signaling switch using multiple autoinhibitory domains [66], the multisite design principle revealed by our model can help guide the engineering of synthetic protein degradation switches, which may have diverse biomedical applications.

## Methods

Systems of ordinary differential equations were formulated and solved with MATLAB 7.1. The codes are available at our website http://www.engin.umich.edu/dept/che/research/lin/downloads.html.

The events integrated in 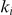 include: binding of ATP and phosphospecies 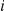 to the kinase, dissociation of phosphospecies 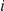 from the kinase, the chemical reaction of phosphorylation and dissociation of the product from the kinase. The events integrated in 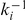 include: binding of ADP and phosphospecies 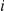 to the phosphatase, dissociation of phosphospecies 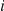 from the phosphatase, the chemical reaction of dephosphorylation and dissociation of the product from the phosphatase. 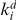 includes all events after phosphorylation to proteolysis.

## Supporting Information

Note S1 Model simplification.
(0.06 MB PDF)

Note S2 Non-dimensionalization.
(0.04 MB PDF)

Note S3 Effect of the sharpness of the stimulus profile.
(0.05 MB PDF)

Figure S1 Change of response coefficient for non-degradable substrate upon single-parameter perturbation.
(20.24 MB TIF)
