# Supplementary material for: Multisite Phosphorylation Provides an Effective and Flexible Mechanism for Switch-Like Protein Degradation
Source: PLoS One. 2010 Dec 13;5(12):e14029. doi: 10.1371/journal.pone.0014029 (PMC3001445; doi:10.1371/journal.pone.0014029)
Supplement: Note S1 — Model simplification. (0.06 MB PDF) [file pone.0014029.s001.pdf]

Supplementary Note S1: Model simplification

In this note, we show that the model equations we have considered in the main text can be derived as an approximation of a complete mechanistic model when
i) the concentration of enzymes are much lower than the initial concentration of the substrate protein; OR
ii) the enzyme-substrate association and dissociation rate constants are much larger than the catalysis rate constants.

First, we assume the reactions for a single-site protein which degrades after phosphorylation follow the mechanistic Michaelis-Menten model:

$$S_0 + kin \underset{k_{off}}{\overset{k_{on}}{\rightleftharpoons}} S_0.kin \xrightarrow{k_{cat}} S_1 + kin$$

$$S_1 + pho \underset{k_{roff}}{\overset{k_{ron}}{\rightleftharpoons}} S_1.pho \xrightarrow{k_{rcat}} S_0 + pho$$

$$S_1 \xrightarrow{k^d} \phi$$

S_0 denotes the unphosphorylated protein which reacts with kinase, denoted by kin. It is assumed that the substrate and kinase react to form an enzyme-substrate complex $S_0.kin$ which can in turn dissociate to form either the enzyme and substrate, or the enzyme and product which is the phosphorylated protein denoted by S_1. Similarly, the phosphorylated protein S_1 and phosphatase, denoted by pho, react to form the substrate-enzyme complex $S_1.pho$ which dissociates to form either the phosphatase and phosphorylated protein or the phosphatase and unphosphorylated protein S_0. For the purposes of this note, we will forget degradation terms in the following calculations. Therefore, the governing equations for this system are as follows:

$$\frac{d[S_0]}{dt} = -k_{on}[kin][S_0] + k_{off}[S_0.kin] + k_{rcat}[S_1.pho] \tag{1a}$$

$$\frac{d[S_1]}{dt} = -k_{ron}[pho][S_1] + k_{roff}[S_1.pho] + k_{cat}[S_0.kin] \tag{1b}$$

$$\frac{d[S_0.kin]}{dt} = +k_{on}[kin][S_0] - k_{off}[S_0.kin] - k_{cat}[S_0.kin] \tag{1c}$$

$$\frac{d[S_1.pho]}{dt} = +k_{ron}[pho][S_1] - k_{roff}[S_1.pho] - k_{rcat}[S_1.pho] \tag{1d}$$

$$[kin] = kin_T - [S_0.kin] \tag{2a}$$

$$[pho] = pho_T - [S_1.pho] \tag{2b}$$

where kin_T and pho_T denote the total concentration of the kinase and phosphatase, respectively; and the last two equations reflect the conservation of mass. We assume that the total concentration of phosphatase is constant, but the total concentration of kinase changes over time. The total concentration of kinase increases non-linearly from an almost zero value to reach a maximum value as follows:

$$kin_T = kin_T^{max} \frac{(t/C)^2}{1 + (t/C)^2} \tag{3}$$

where kin_T^{max} is the maximum that kin_T can reach and C is a constant. The previous model can be extended to a protein that contains n phosphorylation sites and is degraded upon phosphorylation on m sites. In what follows we describe two different hypotheses that reduce the equations described above into the simplified model considered in the main text.

i) Hypothesis regarding the relative amounts of kinase, phosphatase, and substrate

In this section, we show that the complete mechanistic model (described above) can be reduced to the simple model described in the main text when the concentrations of enzymes are much lower than the initial concentration of the substrate protein.

First, we will define dimensionless variables to simplify the analysis: $s_1 = [S_1]/S_T$, where S_T is the total amount of substrate (in the case where degradation is considered, S_T is the initial amount of the substrate); $c = [S_0.kin]/kin_T^{max}$; and $c' = [S_1.pho]/pho_T$. We then define a dimensionless time $\tilde{t} = t\ k_{on} kin_T^{max}$ and the time-derivative with respect to $\tilde{t}$ is denoted by a dot, *i.e.* $\dot{x} = dx/d\tilde{t}$. We also define dimensionless kinase and phosphatase concentrations: $e = [kin]/kin_T^{max}$ and $e' = [pho]/pho_T$. The resulting equations are:

$$\dot{s_1} = -\frac{k_{ron}}{k_{on}} \frac{pho_T}{kin_T^{max}} s_1 e' + \frac{k_{roff}}{k_{on} S_T} \frac{pho_T}{kin_T^{max}} c' + \frac{k_{cat}}{k_{on} S_T} c \tag{4a}$$

$$\dot{c} = \frac{S_T}{kin_T^{max}} (s_0 e - K c) \tag{4b}$$

$$\dot{c'} = \frac{S_T}{kin_T^{max}} \frac{k_{ron}}{k_{on}} (s_1 e' - K' c') \tag{4c}$$

where $K = (k_{off} + k_{cat})/(k_{on} S_T)$ and $K' = (k_{roff} + k_{rcat})/(k_{ron} S_T)$ are normalized Michaelis-Menten constants. We define the parameter $\epsilon = kin_T^{max}/S_T$. In the limit where $\epsilon \to 0$, meaning that $kin_T^{max} << S_T$, and assuming all the kinetic rates are of similar order and kin_T^{max} and pho_T are of similar order too (then implying $kin_T^{max}, pho_T << S_T$), we get a fast dynamics for the complexes c and c' but not for variable s_1 (or s_0, we are just considering one of them here), so that the quasi-steady-state approximation can be applied (with the exception of the initial stage of the reactions). By imposing $\epsilon \dot{c} = 0$ and $\epsilon \dot{c'} = 0$, a little calculation gives:

$$c = s_0 e/K, \qquad c' = s_1 e'/K'. \tag{5}$$

Restoration of dimensions gives the following:

$$[S_0.kin] = \frac{k_{on}}{k_{off} + k_{cat}}[S_0][kin], \qquad [S_1.pho] = \frac{k_{ron}}{k_{roff} + k_{rcat}}[S_1][pho]. \tag{6}$$

Finally, the substitution of these expressions in the equation for S_1 give an equation that depends merely on the concentration of enzymes and free substrate as follows:

$$\frac{d[S_1]}{dt} = -\frac{k_{ron}k_{rcat}}{k_{roff} + k_{rcat}}[pho][S_1] + \frac{k_{on}k_{cat}}{k_{off} + k_{cat}}[kin][S_0] \tag{7}$$

and here we define

$$k_0 = \frac{k_{on}k_{cat}}{k_{off} + k_{cat}} \tag{8a}$$

$$k_0^{-1} = \frac{k_{ron}k_{rcat}}{k_{roff} + k_{rcat}} \tag{8b}$$

Reformulating the equations with the new parameters, and now assuming S_1 is degradable, we obtain:

$$\frac{d[S_0]}{dt} = -k_0[kin][S_0] + k_0^{-1}[pho][S_1] \tag{9a}$$

$$\frac{d[S_1]}{dt} = -k_0^{-1}[pho][S_1] + k_0[kin][S_0] - k^d[S_1] \tag{9b}$$

which are the equations that we use throughout the paper.

The described condition, that the concentrations of enzymes are much lower than the concentration of substrate, may be satisfied for the majority of *in vitro* assays. However, it may be too restrictive under other conditions. Therefore, we have found another condition, described in the following section.

ii) Hypothesis regarding the kinetic rates

In this section, we show that the complete mechanistic model can be replaced by a reduced simplified one when the enzyme-substrate association and dissociation rate constants are much larger than the catalysis rate constants, and hence the enzyme-substrate complexes evolve in a fast time scale compared with the substrates.

First, we define a new variable X in such a way that $X = [S_1] + [S_1.pho]$. X satisfies the following differential equation:

$$\frac{d[X]}{dt} = k_{cat}[S_0.kin] - k_{rcat}[S_1.pho] \tag{10a}$$

The dimensionless form of X is $x = s_1 + c'(pho_T/S_T)$ and then the system of equations becomes:

$$\frac{dx}{dt} = k_{cat}(kin_T^{max}/S_T)c - k_{rcat}(pho_T/S_T)c' \tag{11a}$$

$$\frac{dc}{dt} = (k_{on}S_T)s_0e - (k_{off} + k_{cat})c \tag{11b}$$

$$\frac{dc'}{dt} = (k_{ron}S_T)s_1e' - (k_{roff} + k_{rcat})c' \tag{11c}$$

We now consider that the rates of association and dissociation between the enzyme and the substrate are much higher than the rate of product formation and dissociation: $k_{on}S_T$, $k_{ron}S_T$, k_{off}, $k_{roff} >> k_{cat}$, k_{rcat}. In this case, the above equations for c and c' have fast rates in both of their two terms, while the equation for x has slow rates in both of its two terms. Under these conditions the quasi-steady-state approximation can be applied, which leads to the same expressions we got in Hypothesis A) for c and c'. Therefore, we can again obtain the simplified model used in the main text.
