# Supplementary material for: Multisite Phosphorylation Provides an Effective and Flexible Mechanism for Switch-Like Protein Degradation
Source: PLoS One. 2010 Dec 13;5(12):e14029. doi: 10.1371/journal.pone.0014029 (PMC3001445; doi:10.1371/journal.pone.0014029)
Supplement: Note S2 — Non-dimensionalization. (0.04 MB PDF) [file pone.0014029.s002.pdf]

Supplementary Note S2: Non-dimensionalization

In this note, we describe how the ODE system presented in the main text is non-dimensionalized to simplify the analysis. As described in the main text, the substrate protein contains n sites and degrades after being phosphorylated on m sites. The concentration of each phospho-state is a function of the concentrations of the upstream and downstream phospho-states, the kinase and phosphatase, and the kinetic parameters. For illustration purpose, consider the governing equations for the sequential system.

$$\frac{d[S_0]}{dt} = k_0^{-1}[pho][S_1] - k_0[kin][S_0] - k_0^d[S_0] \tag{1a}$$

$$\frac{d[S_i]}{dt} = k_{i-1}[kin][S_{i-1}] + k_i^{-1}[pho][S_{i+1}] - k_i[kin][S_i] - k_{i-1}^{-1}[pho][S_i] - k_i^d[S_i] \quad i = 1, ..., n-1 \tag{1b}$$

$$\frac{d[S_n]}{dt} = k_{n-1}[kin][S_{n-1}] - k_{n-1}^{-1}[pho][S_n] - k_n^d[S_n] \tag{1c}$$

where k_i, k_i^{-1} and k_i^d stand for kinetic rate constants of phosphorylation, dephosphorylation and degradation, respectively. $[kin]$, $[pho]$ and $[S_i]$ represent concentrations of the kinase, phosphatase and the substrate with i sites phosphorylated, respectively. $k_i^d = 0$ for $i = 0, ..., m-1$ and $k_i^d \neq 0$ for $i = m, ..., n$.

Let us look at the general case represented by Eq. (**??**). To non-dimensionalize this equation, we use the initial concentration of the substrate, S_{Ini}, to normalize the dependent variable $[S_i]$:

$$[\bar{S_i}] \equiv \frac{[S_i]}{S_{Ini}}. \tag{2}$$

For non-dimensionalization of the independent variable t, let us choose the time scale associated with the degradation reaction.

$$\bar{t} \equiv \frac{t}{1/k_{ave}^d} \tag{3}$$

where k_{ave}^d is average of the degradation rate constants:

$$k_{ave}^d = \frac{1}{n+1-m}\sum_{i=m}^{n} k_i^d. \tag{4}$$

Substituting $[S_i]$ and t with the new set of dimensionless variables $[\bar{S_i}]$ and $\bar{t}$ gives the following non-dimensionalized equation:

$$\frac{d[\bar{S_i}]}{d\bar{t}} = \frac{k_{i-1}}{k_{ave}^d}[kin][\bar{S}_{i-1}] + \frac{k_i^{-1}}{k_{ave}^d}[pho][\bar{S}_{i+1}] - \frac{k_i}{k_{ave}^d}[kin][\bar{S_i}] - \frac{k_{i-1}^{-1}}{k_{ave}^d}[pho][\bar{S_i}] - \frac{k_i^d}{k_{ave}^d}[\bar{S_i}]. \tag{5}$$

Now we define three sets of new dimensionless parameters:

$$\alpha_i \equiv \frac{k_i}{k_{ave}^d}[kin] \tag{6a}$$

$$\beta_i \equiv \frac{k_i^{-1}}{k_{ave}^d}[pho] \tag{6b}$$

$$\gamma_i \equiv \frac{k_i^d}{k_{ave}^d} \tag{6c}$$

and eventually end up with

$$\frac{d[\bar{S}_i]}{d\bar{t}} = \alpha_{i-1}[\bar{S}_{i-1}] + \beta_i[\bar{S}_{i+1}] - \alpha_i[\bar{S}_i] - \beta_{i-1}[\bar{S}_i] - \gamma_i[\bar{S}_i]. \tag{7}$$

In this work, we assume that the degradation rate constants are the same for all the phospho-states with m or more sites phosphorylated. In this case, the system reduces from five types of parameters in the original system $(k_i, k_i^{-1}, k_i^d, [kin], [pho])$ to two sets in the dimensionless space (α_i, β_i). Equivalently, we can simply set the degradation rate constants k_i^d to 1 and change $k_i[kin]$ and $k_i^{-1}[pho]$ as whole groups in the original system when exploring the effect of parameters.

It should also be noted that the concentration of kinase may change over time and also needs to be expressed as a function of the dimensionless time while we work in the non-dimensionalized space:

$$[kin] = f(t) = \bar{f}(\bar{t}). \tag{8}$$
