# Supplementary material for: Multisite Phosphorylation Provides an Effective and Flexible Mechanism for Switch-Like Protein Degradation
Source: PLoS One. 2010 Dec 13;5(12):e14029. doi: 10.1371/journal.pone.0014029 (PMC3001445; doi:10.1371/journal.pone.0014029)
Supplement: Note S3 — Effect of the sharpness of the stimulus profile. (0.05 MB PDF) [file pone.0014029.s003.pdf]

Supplementary Note S3: Effect of the sharpness of the stimulus profile

To model the kinase stimulus, we explored three different types of profiles: step function, linear, and sigmoid. For sigmoid kinase stimuli, we further examined the effect of the sharpness of the stimulus profile on the steepness of the response curve, particularly of the non-degradable substrate. As illustrated in Table S1, for $n = 9$ and $m = 5$, increasing the nonlinearity of the sigmoid stimulus does not lead to significant improvement of the steepness of the response curve when the exponent of t is greater than two.

Table S1. Response coefficients for the non-degradable amount of the substrate ($n = 9$)†

	$m = 1$			$m = 5$			$m = 9$		
$Kin(t)$	$R_{0.5/0.9}$	$R_{0.1/0.5}$	$R_{0.1/0.9}$	$R_{0.5/0.9}$	$R_{0.1/0.5}$	$R_{0.1/0.9}$	$R_{0.5/0.9}$	$R_{0.1/0.5}$	$R_{0.1/0.9}$
5	7.00	3.71	26.00	2.00	1.82	3.64	1.70	2.08	3.54
$5t$	2.90	2.00	5.80	1.48	1.35	2.00	1.31	1.60	2.10
$5\frac{t}{1+t}$	3.00	2.13	6.40	1.54	1.44	2.21	1.40	2.13	2.98
$5\frac{t^2}{1+t^2}$	2.00	1.68	3.35	1.31	1.27	1.67	1.27	1.62	2.05
$5\frac{t^4}{1+t^4}$	1.53	1.32	2.02	1.18	1.16	1.37	1.15	1.34	1.54
$5\frac{t^{10}}{1+t^{10}}$	1.20	1.14	1.38	1.07	1.08	1.16	1.07	1.15	1.24
$5\frac{t^{20}}{1+t^{20}}$	1.10	1.07	1.19	1.05	1.06	1.11	1.05	1.11	1.16

†All phosphorylation and dephosphorylation rate constants are 10 and 20, respectively. The degradation rate constant is 1 for all degradable phospho-states.
