# Supplementary figures and images for: Multisite Phosphorylation Provides an Effective and Flexible Mechanism for Switch-Like Protein Degradation
Source: PLoS One. 2010 Dec 13;5(12):e14029. doi: 10.1371/journal.pone.0014029 (PMC3001445; doi:10.1371/journal.pone.0014029)

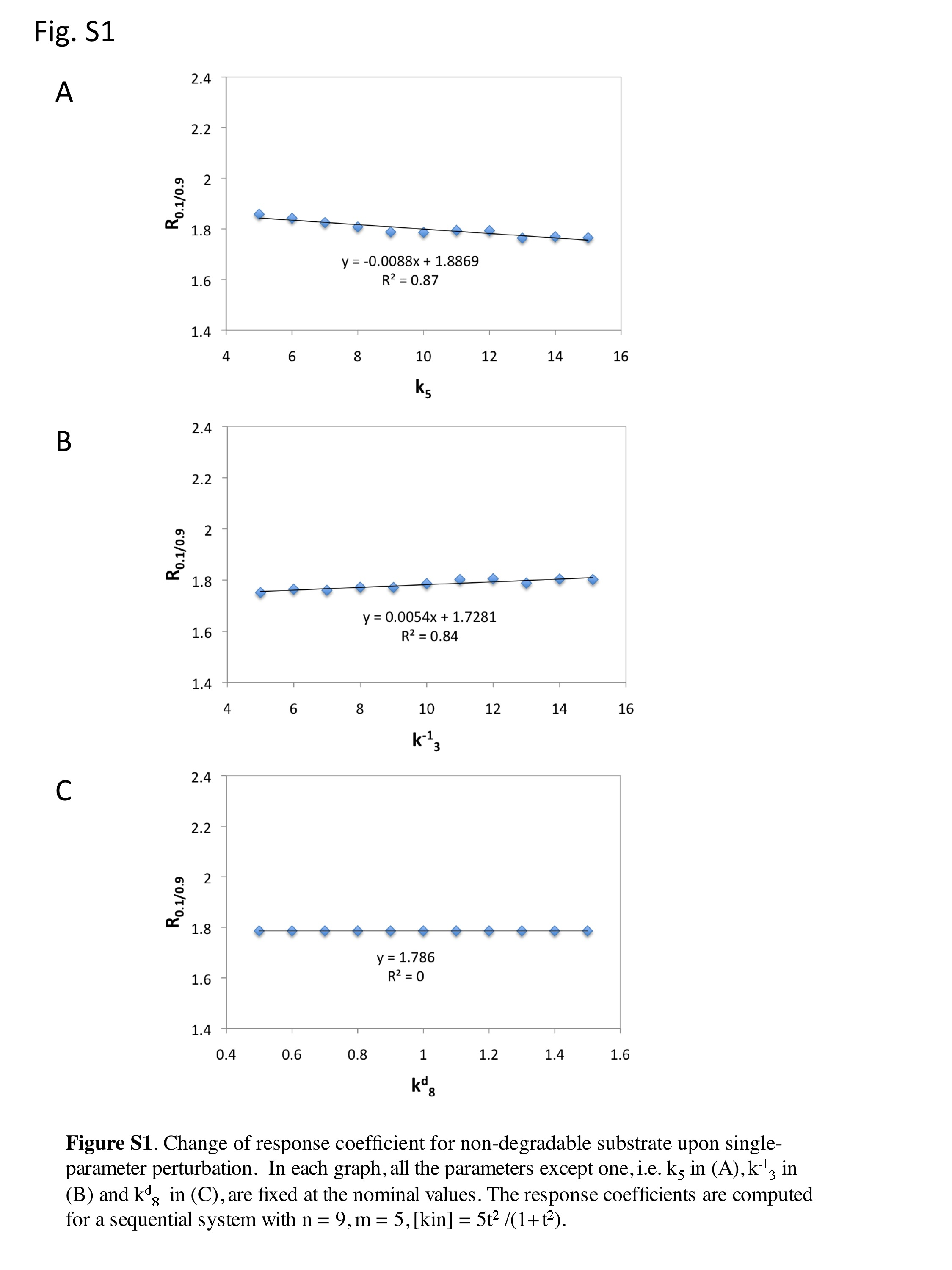

Supplement: Figure S1 — Change of response coefficient for non-degradable substrate upon single-parameter perturbation. (20.24 MB TIF) [file pone.0014029.s004.tif]
